# Overexpression of Pericentromeric HSAT2 DNA Increases Expression of EMT Markers in Human Epithelial Cancer Cell Lines

**DOI:** 10.3390/ijms24086918

**Published:** 2023-04-07

**Authors:** Nikita Ponomartsev, Danil Zilov, Ekaterina Gushcha, Alexandra Travina, Alexander Sergeev, Natella Enukashvily

**Affiliations:** 1 Institute of Cytology, Russian Academy of Sciences, St. Petersburg 194064, Russia; 2 Applied Genomics Laboratory, SCAMT Institute, ITMO University, Saint Petersburg 191002, Russia

**Keywords:** pericentromere, satellite DNA, human satellite 3 transcription, long noncoding RNA, CHM13 assembly

## Abstract

Pericentromeric tandemly repeated DNA of human satellites 1, 2, and 3 (HS1, HS2, and HS3) is actively transcribed in some cells. However, the functionality of the transcription remains obscure. Studies in this area have been hampered by the absence of a gapless genome assembly. The aim of our study was to map a transcript that we have previously described as HS2/HS3 on chromosomes using a newly published gapless genome assembly T2T-CHM13, and create a plasmid overexpressing the transcript to assess the influence of HS2/HS3 transcription on cancer cells. We report here that the sequence of the transcript is tandemly repeated on nine chromosomes (1, 2, 7, 9, 10, 16, 17, 22, and Y). A detailed analysis of its genomic localization and annotation in the T2T-CHM13 assembly revealed that the sequence belonged to HSAT2 (HS2) but not to the HS3 family of tandemly repeated DNA. The transcript was found on both strands of HSAT2 arrays. The overexpression of the HSAT2 transcript increased the transcription of the genes encoding the proteins involved in the epithelial-to-mesenchymal transition, EMT (*SNAI1*, *ZEB1*, and *SNAI2*), and the genes that mark cancer-associated fibroblasts (*VIM*, *COL1A1*, *COL11A1*, and *ACTA2*) in cancer cell lines A549 and HeLa. Co-transfection of the overexpression plasmid and antisense nucleotides eliminated the transcription of EMT genes observed after HSAT2 overexpression. Antisense oligonucleotides also decreased transcription of the EMT genes induced by tumor growth factor beta 1 (TGFβ1). Thus, our study suggests HSAT2 lncRNA transcribed from the pericentromeric tandemly repeated DNA is involved in EMT regulation in cancer cells.

## 1. Introduction

More than 90% of a mammalian genome is composed of repetitive sequences. The repetitive sequences can be divided into two families: interspersed repeats and tandem repetitive (TR) sequences. TR consist of a sequentially repeated motif in one of the two possible orientations: head-to-tail (direct repeats) or head-to-head (inverted repeats) [1]. Tandemly repeated DNA (TR DNA) is estimated at approximately 10% of the human genome. The family includes tandem gene paralogues, rDNA, and satellite DNA that comprises big or classical satellites, minisatellites, and microsatellites. Originally, big satellites were referred to as human satellites I, II, and III [2] according to the position of bands in the cesium density gradient. Later, it was suggested to name the specific repetitive DNA families within each satellite fraction using Arabic numerals, while the cesium density gradient satellite fractions retained their Roman numerals [3]. Human satellites 2 and 3 define two related families with a frequent motif GGAAT (ATTCC) which is now referred to more often as CATTC [4,5]. Human satellite 3 (HSAT3, referred to as HS3 in early publications) contains two types of repeat units: a 5 bp imperfect GGAAT (ATTCC; most pentamers are sequence variants such as CCAGT, CGATT, and CCGCT) repeat and a 10 bp CAACCCGAGT repeat [3,6]. The same GGAAT pentamer was the origin of human satellite 2 DNA (HSAT2, previously referred to as HS2). However, HSAT3 shows a strict periodicity of 5 bp, and HSAT2 is built on two tandemly repeated ATTCCATTCG and one or two ATG repeats with a consensus of 23 and 26 bp [3,4,7]. The GATGAT motif features HS3 of the 1qh region [8] (the band nomenclature is given according to the International System for Human Cytogenomic Nomenclature [9]. ‘1qh’ denotes ‘heterochromatin pericentromeric region on chromosome 1 q-arm’).

At the end of the last century, the first data appeared on the role of classical satellites. Their transcription was shown in different organisms [10,11,12]. The number of satellite transcripts 2 and 3 increases manyfold at embryogenesis, cell differentiation, at the beginning of proliferation, cell aging, carcinogenesis, and cell stress [13,14,15,16].

Earlier, we detected HS2/HS3 transcription in human preovulatory oocytes [17,18], some cancer cell lines, and senescent and cancer-associated fibroblasts [13,19]. At that time, mapping the sequence onto human chromosomes and analysis of adjacent chromosomal regions was complicated because pericentromeric and centromeric TR DNA sequences remained almost entirely missing from the assembled human reference genome for the past 20 years [20]. The majority of these sequences were also not annotated. Therefore, reliable analysis of sequence position in the genome or their presence in transcriptome was impossible. However, in 2022, the T2T-CHM13 assembly was published [21] and become available in Genome Browser (https://genome.ucsc.edu/cgi-bin/hgGateway, last accessed on 28 March 2023) [22] and GenBank (https://www.ncbi.nlm.nih.gov/data-hub/genome/GCF_009914755.1/, last accessed on 28 March 2023) [23]. The new assembly includes gapless assemblies for all chromosomes except Y and introduces nearly 200 million base pairs of sequences containing 1956 genetically generated models of alpha satellite predictions, 99 of which are predicted to be protein-coding. In T2T-CHM13 assembly, for the first time in the genome era, pericentromeric and centromeric repeats, which constitute 6.2% of the genome (189.9 megabases) were fully characterized and the gapless maps of pericentromeres were provided [24].

The absence of pericentromeric region maps [20] made it difficult to study the functions of the long noncoding RNA (lncRNA) transcribed from pericentromeres. The publication of the gapless T2T-CHM13 assembly allowed us to continue our study of the HS2/HS3 transcription. It provided us with a useful tool for the analysis of chromosomal localization and engineering a plasmid overexpressing the HS2/HS3 transcript we had described earlier. The plasmid overexpressing HS2/HS3 was then used for functional studies on cancer cells. Transcription of TR DNA of human pericentromeric satellites was demonstrated in solid tumors and in hematological malignancies [19,25,26,27], where it was transcribed by cancer cells [25] and/or by microenvironment cells [19,27]. The association of HS2/HS3 transcription with a poor prognosis has been demonstrated [26]. However, the mechanisms underlying this association remain unclear.

The aim of our study was to map a previously described HS2/HS3 transcript on chromosomes using a gapless genome assembly, verify it, and create a plasmid overexpressing the transcript to assess the influence of HS2/HS3 transcription on cancer cells.

We report here that the sequence of the previously identified transcript is tandemly repeated on nine chromosomes (1, 2, 7, 9, 10, 16, 17, 22, and Y). Despite our previous description of it as human satellite III or human satellite 2/3 (HS2/HS3) transcript [13,17,19,27], a detailed analysis of its genomic localization and annotation in the T2T-CHM13 assembly revealed that the sequence belonged to the HSAT2 (human satellite 2) family of TR DNA. Unexpectedly the transcript DNA was found on both strands of HSAT2 arrays. This finding introduces some doubt regarding the general opinion about the strand-specificity of HSAT transcription—the methods used in these works did not take into account the possibility of HSAT inversions. The overexpression of the HSAT2 transcript increased the transcription of genes encoding the proteins involved in the epithelial-to-mesenchymal transition, EMT (Snail, Zeb1, Slug), and the proteins that mark mesenchymal cells with cancer-associated fibroblast phenotypes (vimentin, collagens 1A1, 11A1, and alpha-smooth muscle actin) in cancer cell lines A549 and HeLa. Co-transfection of the overexpression plasmid and antisense nucleotides eliminated the transcription of EMT genes. Antisense oligonucleotides decreased the transcription of the EMT genes induced by tumor growth factor beta 1 (TGFβ1). Thus, our study suggests HSAT2 lncRNA transcribed from the pericentromeric TR DNA is involved in EMT regulation in cancer cells.

## 2. Results

### 2.1. Chromosomal Localization of Transcribed TR Satellite DNA

In our previous work, four transcripts were found in human oocyte transcriptomes [17]. However, they were not mapped onto chromosomes due to the absence of gapless genome assembly. The transcripts were quantified. One of them (available in the Third Party Annotation Section of the DDBJ/ENA/GenBank databases under the accession number TPA: BK063198) was accessed as the most abundant and was chosen for further studies. It was shown to share a high degree of homology with the transcript (GenBank acc. no. X60726.1) we had described earlier in the human embryo, senescent, and cancer cells [13,28]. However, we could not study the exact position of the DNA encoding the transcript using in silico methods because some of the repeats residing within the regions were omitted in the assemblies available at that time (GRCh38/hg38 and earlier), though fluorescence in situ hybridization (FISH) analysis proved its position at least on chromosome 1 [13].

**Figure 1:**
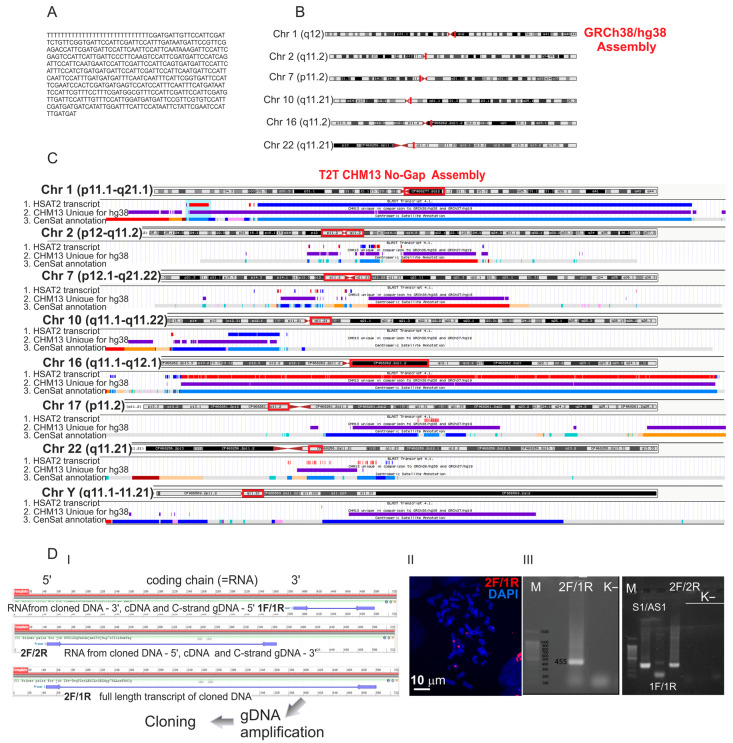
Mapping the transcript (Acc No TPA: BK063198): the sequence (**A**) annotated in [17] in the GRCh38/hg38 (**B**) and T2T-CHM13 (**C**) assemblies. (**B**) Localization of the transcript is shown as red lines on chromosome ideograms. (**C**) The transcript mapping is visualized in Genome Browser using the bed-files given in the Supplementary Materials (Supplementary File S1). Three tracks are shown: 1—the transcript track (red and blue—“+” and “–“ orientation of the repeated arrays of the sequence hits, respectively), 2—the track of sequences unique to T2T-CHM13 as compared to GRCh38/hg38, and 3—the track of satellite annotation in the T2T-CHM13 assembly. The colors in the third track are given according to the Centromeric Satellite Annotation colors in UCSC Genome browser. Active αSat HOR (hor … L)—red, inactive αSat HOR (hor)—orange, divergent αSat HOR (dhor)—dark red, monomeric αSat (mon)—peach/yellow, classical Human Satellite 1A (HSAT1A)—light green, Classical Human Satellite 1B (HSAT1B)—dark green, Classical Human Satellite 2 (hsat2)—light blue, Classical Human Satellite 3 (HSAT3)—blue, Beta Satellite (bsat)—pink, Gamma Satellite (gsat)—purple, other centromeric satellites (censat)—teal, centromeric transition regions (ct)—grey, classical Human Satellite 2 (HSAT2)—light blue. The transcript hits (red and blue in track 1) are positioned within HSAT2 arrays (light blue in track 3). Other colors for track 3 are shown at the link. (**D**) Panel I—Three sets of primers were designed to amplify from genomic DNA: 5′ (2F/2R primers in Table 2), 3′ (1F/1R) parts, and the full length of the sequence (2F/1R primers pair). Panel II—2F/1R was Cy3 labeled by PCR and used as a FISH probe to verify its pericentromeric localization in mesenchymal stromal cells obtained as described in Supplementary Figure S1). Panel III—PCR of genomic DNA with 2F/1R, 1f/1R, 2F/2R, and S1/AS1 primers. K^−^—NTC control, M—marker, S1/AS1—HSAT2 amplificated with the primers we described in [13].

In the present study, we first mapped the sequence (Figure 1A) onto chromosomes using both the GRCh38/hg38 and T2T-CHM13 assemblies (Figure 1B,C). The latter assembly contained many more hits for the transcript sequence as compared to GRCh38/hg38. The greatest number of hits (7876 of 12,262) with the highest degree (96.93–97.15%) of homology was detected in the chromosome 1 sequence (Figure 1C and Table 1). This result matched the FISH data from our previous work [13]. Additionally, the tandem arrays of the sequence were also present on chromosome 16. However, TR DNA homologous to the transcript was also found on chromosomes 2, 7, 9, 10, 17, 22, and Y (Figure 1B,C and Table 1; Supplementary Table S1 and File S1). Notably, in all chromosomes, most of the hits resided within the sequences unique for the T2T-CHM13 assembly that were absent in GRCh38/hg38 (Figure 1C, tracks ‘transcript 4.1’ and ‘CHM13 unique in comparison to GRCh38/hg38’). Therefore, before the publication of the T2T-CHM13 assembly, precise mapping and analysis of HSAT sequences including the sequences we described were hardly possible.

In all the chromosomes, the TR DNA homologous to the transcript was positioned only in pericentromeric satellite DNA arrays. All the hits were mapped within different subfamilies of HSAT2 but not HSAT3 (Table 1, and Figure 1C; Supplementary Table S1 and File S1) despite the annotation as HSAT3 or HSAT2/3 in our previous studies [8,13,17].

**Figure 2:**
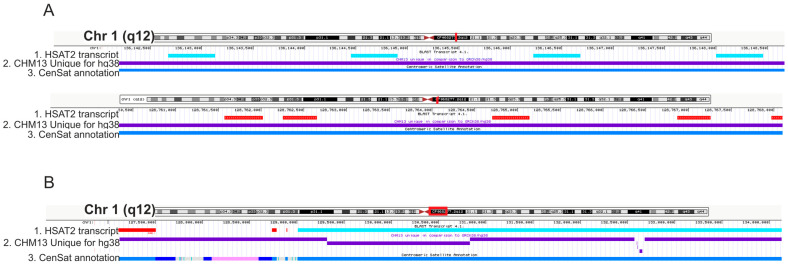
“+” and “−” orientation of the cloned transcript hits arrays. (**A**) Visualization of 2 fragments of chromosome 1 pericentromeric region (red lines on chromosome 1 ideograms) at high magnification. Turquoise rectangles in track 1—HSAT2 transcript arrays in the “−“ (G-rich) strand. Red rectangles in track 1—HSAT2 transcript arrays in the “+” (C-rich) strand. (**B**) Chromosome 1 pericentromeric region at low magnification (red frame on chromosome 1 ideogram). Track 1 shows hits in the opposite direction (red and turquoise rectangles) that compose spatially separated tandemly organized arrays. Within each array, repeating units of the transcript are positioned in a head-to-tail manner. Track 2 in (**A**,**B**)—the track of sequences (purple rectangles) unique to T2T-CHM13 as compared to GRCh38/hg38; track 3—the track of satellite annotation. The colors in the third track are shown according to the Centromeric Satellite Annotation colors given in UCSC Genome browser. Active αSat HOR (hor … L)—red, inactive αSat HOR (hor)—orange, divergent αSat HOR (dhor)—dark red, monomeric αSat (mon)—peach/yellow, classical Human Satellite 1A (HSAT1A)—light green, Classical Human Satellite 1B (HSAT1B)—dark green, Classical Human Satellite 2 (hsat2)—light blue, Classical Human Satellite 3 (HSAT3)—blue, Beta Satellite (bsat)—pink, Gamma Satellite (gsat)—purple, other centromeric satellites (censat)—teal, centromeric transition regions (ct)—grey, classical Human Satellite 2 (HSAT2)—light blue.

Unexpectedly, the orientation of the DNA coding the transcript varied between chromosomes, between subfamilies in a chromosome, and even within a subfamily (Figure 1C and Figure 2A,B, and Table 1). The transcript sequence used for the study was taken from a transcriptome i.e., it was cDNA. That means that in chromosome 1 most of the hits were positioned in one orientation (on the C or +-strand because cDNA complementary to RNA is positioned on the G-strand) while in chromosomes 17, 9, and 22—in the opposite direction. In chromosomes 2, 7, and 16, the homologous DNA was found on both strands even within a subfamily (Table 1).

### 2.2. Designing a Plasmid Overexpressing HSAT2

We selected three sets of primers (Table 2 and Figure 1D). Primer set 2F/1R covers the entire sequence (Acc No TPA: BK063198) shown in Figure 1A amplifying a sequence of 455 bp (Figure 1D panel I) that hybridizes exclusively to the pericentromeric regions of chromosomes (Figure 1D and Supplementary Figure S1). Primer set 1F/1R amplified the 3′ end of the transcript (i.e., 5′ end of the cDNA obtained from the RNA expressed in the plasmid). Primers 2F/2R were complementary to the 5′ part of the RNA/3′ part of cDNA.

**Figure 3:**
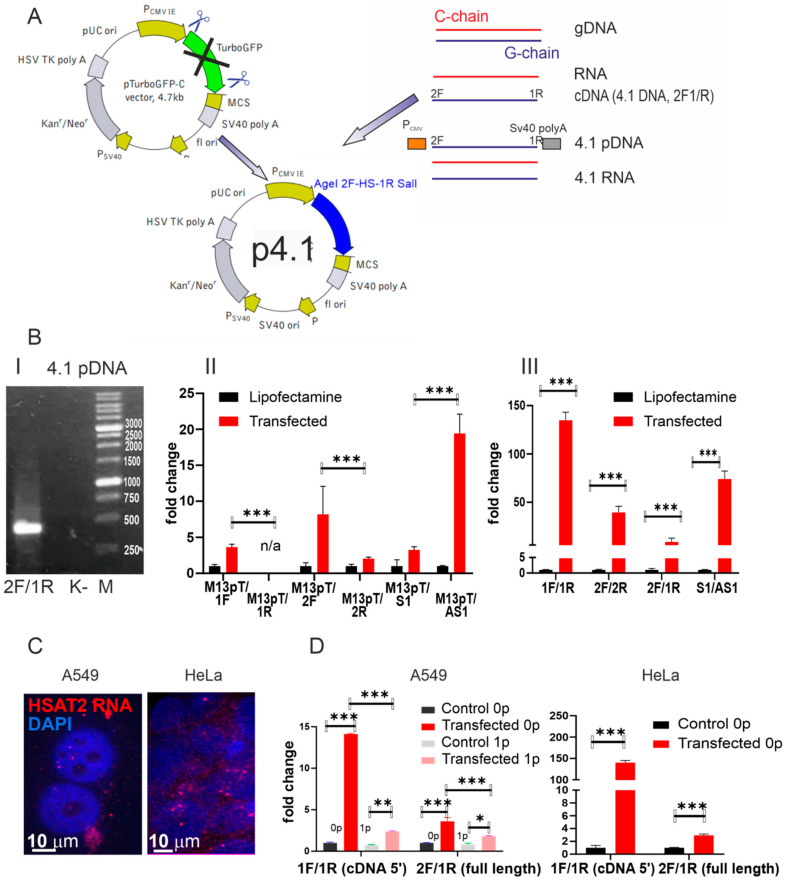
Genetic construct for HSAT2 (Acc No OP912406.2) overexpression. (**A**) Scheme of cloning (see the Materials and Methods Section 4 for details). (**B**) Panel I—OP912406.2 amplification with 2F/1R primers using p4.1 plasmid as a template. K−—NTC control; M—molecular weights (bp) marker (Sibenzyme, Russia). Panel II—qPCR quantification of A549 M13-oligo-dT cDNA (see Section 4.6 of Materials and Methods) with a different set of primers (see Table 2 for primer sequences and abbreviations) to prove (a) the amplified cDNA originated from the cloned sequence but not from genomic DNA and (b) it can be amplified with the S1/AS1 primer pair from our previous work. Panel III—qPCR with different primers set to quantify different parts of the transcript cDNA (1F/1R—5′ of cDNA; 2F/2R—3′ of cDNA; 2F/1R—the whole transcript; S1/AS1—amplification with previously described primers to prove the sequence homology with the previously described transcript). (**C**) DNA-RNA FISH of transfected A549 and HeLa cells. HSAT2 probe is shown in red, nuclei are counterstained with DAPI (blue), and scale bars are shown in the images. (**D**) Quantification of 4.1 (Acc No OP912406.2) transcription before and after the first splitting. *—*p* < 0.05, **—*p* < 0.01, and ***—*p* < 0.001.

A modified 2F/1R primer set was used to amplify genomic DNA that was further cloned into pTurboGFP-c vector where GFP was substituted with a cloned sequence (Figure 3A) as described in the Materials and Methods Section 4. 2F/1R primers were modified for cloning: 2F primer was combined with a fragment of the AgeI site of restriction at its 5′ end while a part of the SalI site of restriction was added to the 1R primer 5′ end (Table 2). Therefore, the inserted sequence was oriented to obtain a transcript identical to the sequence shown in Figure 1A (i.e., it was a coding strand of the plasmid). Five clones were obtained after the transformation of the *Escherichia coli* DH5α strand (GenBank acc. nos. OP912403.2, OP912404.2, OP912405.2, OP912406.2, and OP912407.2). The cloned sequence alignment is shown in Supplementary Figure S2. They shared a high degree of homology between each other and the transcribed sequence (Acc No TPA: BK063198) we identified previously. The clone 4.1 (GenBank Acc No OP912406.2) was chosen for further studies as a sequence with the highest homology to the transcribed sequence we described earlier.

The vector pTurboGFP-C retained a cytomegalovirus (CMV) promoter and a polyadenylation signal after our modification (Figure 3A). Thus, the sequence inserted instead of Turbo-GFP should be constitutively expressed and polyadenilated in transfected cells. It was confirmed with a quantitative PCR of M13poliT cDNA of transfected cells using either M13 primer along with one of the primers: 2F, 1F, 2R, and 1R primer or 1F/1R, 2F/2R, and 2F/1R primer pairs (Figure 3B). The amplicon quantity was significantly higher when a forward primer was added to the mix along with M13 as compared to the corresponding reverse primer. Notably, the primer set S1/AS1 could also amplify the plasmid transcripts. The set was designed during our previous work ([13], Figure 10c of the cited work) to amplify transcripts of satellite DNA in senescent and some cancer cells. We demonstrated that the transcript amplified with S1/AS1 primers was a part of the transcript revealed later ([17], Figure 6c of the cited work) and used in the current study as a matrix for 2F/1R primers (Figure 1A). However, in the study carried out in 2007, the orientation was different—amplification was observed by routine PCR when M13/S1 was used while in the current study, the qPCR amplification was higher when the M13poliT/AS1 pair was added to the mix (Figure 3B). It corresponds to the results we report here—the sequence shown in Figure 1A (obtained from a transcriptome i.e., cDNA) and used as a matrix for 1F, 1R, 2F, and 2R primer design is abundant on chromosome 1 though oriented in the opposite direction (Table 1) to the C-strand. S1/AS1 set was designed using the information about the chromosome 1 specific C-strand TR gDNA (acc. no. X60726) that was available at that time.

In situ, the transcript was detected both in the cytoplasm and nucleoplasm 72 h post-transfection (Figure 3C).

The transfection was effective in two cancer cell lines of epithelial origin, A549 and HeLa—the transcriptions increased 15–140-fold after transfection (Figure 3D). However, the transcription that appeared after transfection declined rapidly during cell culture proliferation (Figure 3D). After the first splitting of the culture, the fold change ratio between transfected and non-transfected samples decreased from 4.1 ± 0.44 to 1.4 ± 0.08. It should also be noted that the HSAT2 transcription fold change ratio between transfected and mock-transfected cells was always higher when the 5′ end of the cDNA was amplified (Figure 3D) as compared to whole transcript amplification. We assume this is due to the rapid RNA 3′ end degradation.

The genetic construct we assembled was used in further studies on the functional significance of the HSAT2 transcription.

### 2.3. Influence of the Overexpression on Epithelial–Mesenchymal Transition

**Figure 4:**
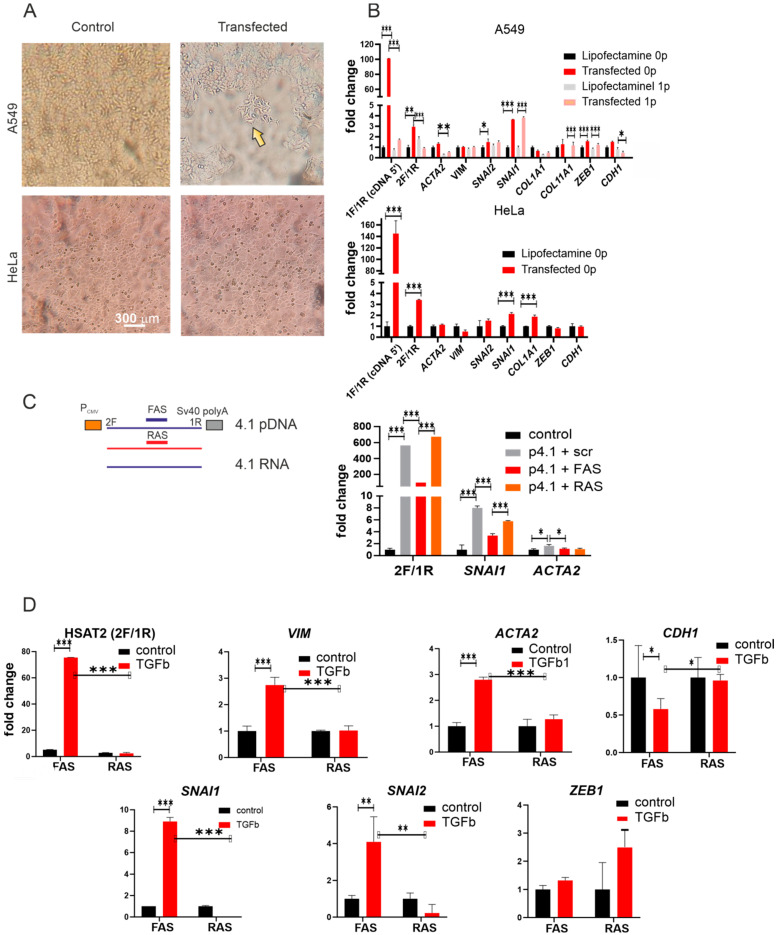
HSAT2 transcription influence on EMT. (**A**) Morphology of A549 and HeLA cell cultures transfected with 4.1 plasmid. Arrow—cells with non-epithelial morphology. (**B**) Quantification of EMT marker gene transcription in transfected A549 and HeLa cells. 0 p—transfected cells before the first splitting; 1p—after the first splitting. (**C**) Knockdown of 4.1 (Acc No OP912406.2) transcripts in transfected A549 cells. The left image shows a scheme of FAS and RAS 2-o-Me/LNA-modified oligonucleotides annealing on 4.1 sequence strands, CMV—cytomegalovirus promoter in the plasmid; 2F, 1R—the primer position (see Table 2 for primer sequences and abbreviations). Right image—quantification of 4.1 transcript in cells co-transfected with the overexpression plasmid (p4.1) and either scramble (scr), FAR, or RAS—2-o-Me/LNA-modified oligonucleotides; control—cells treated with lipofectamine only (without DNA). (**D**) Influence of FAS and RAS antisense oligonucleotides on EMT markers’ mRNA level in A549 cells treated with TGFβ1 to induce EMT. The abbreviations are the same as in C. See Table 2 for the primer sequences and targets. *—*p* < 0.05, **—*p* < 0.01, and ***—*p* < 0.001.

During visual inspection of transfected A549 cells, we noticed that the number of cells of non-epithelial morphology increased as compared to control cells treated with lipofectamine (Figure 4A). Therefore, we accessed the transcription of ‘master’ EMT-inducing genes (*SNAI1*, *ZEB1*, and *SNAI2*) encoding proteins involved in the process of EMT (Snail, Zeb1, and Slug) [33]. We also accessed markers of the mesenchymal phenotype (vimentin, collagens 1A1, 11A1, and alpha-smooth muscle actin) in the A549 cell line. Mesenchymal cells express little or no E-cadherin while epithelial cells are known for their high level of the protein and its mRNA [34]. We also added the HeLa cell line to the study to check whether the effect of transfection on EMT could be observed in other epithelial cancers where changes in morphology after transfection was not so clearly visible (Figure 4A).

EMT marker genes were activated after transfection of A549, albeit to a different extent (Figure 4B). The highest level of upregulation was observed for the *SNAI1* gene encoding the Snail protein. The upregulation persisted through the passaging of cell culture (fold change: 3.69 ± 0.02 at passage 0 vs. 3.82 ± 0.1 after the first passage of the transfected cells). Transcription of the *SNAI2* gene encoding the Slug protein was also increased but to a lesser extent (1.50 ± 0.3 at passage 0 vs. 1.47 ± 0.15 at passage 1). *ZEB1* transcription changed in a similar way (1.58 ± 0.11 at passage 0 vs. 1.23 ± 0.14). EMT marker transcription was also activated in transfected HeLa cells (Figure 4B). However, the fold change ratio confirmed the weaker activation of EMT marker genes in HeLa than in A549. *SNAI1* transcription was only 2.13 ± 0.13-fold increased 48 h after transfection; SNAI2—1.52 ± 0.5-fold. *ZEB1* transcription was not activated in HeLa cells after transfection.

Cells after EMT acquire the mesenchymal phenotype: they exhibited high levels of- vimentin, collagens 1A1, 11A1, alpha-smooth muscle actin, and low E-cadherin mRNAs. *CDH1* (E-cadherin) transcription was strongly downregulated in A549 cells but not in HeLa cells (Figure 4B). However, co-transfection with the HSAT2-expressing plasmid and an LNA-modified oligonucleotide (FAS) complementary to the coding chain of the plasmid (i.e., to plasmid RNA) decreased both HSAT2 (from 564.9 ± 0.3 to 97.4-fold change vs. untreated cells) and *SNAI1* (from 8.35 ± 0.3 to 3.35 ± 0.32-fold change) transcription and returned ACTA2 mRNA quantity to its level in untreated cells (from 1.65 ± 0.22 to 1.14 ± 0.12) (Figure 4C). An LNA-modified oligonucleotide (RAS) was complementary to the non-transcribed chain of the plasmid.

### 2.4. Knockdown of the HSAT2 Transcript Decreased TGFβ1-Induced Upregulation of EMT Gene Transcription

Our study gave evidence that HSAT2 overexpression induced EMT transition in two cancer cell lines. In the next step, we addressed the question of how HSAT2 knockdown would influence the EMT gene activation induced by TGFβ1.

In A549 cells treated with TGFβ1, HSAT2 transcription was strongly upregulated (75.2 ± 0.2-fold change vs. cells untreated with the growth factor) (Figure 4D). Such a powerful increase in the expression level is explained by the fact that the sequence amplified by 2F/1R primers (Table 2, Figure 1D and Figure 2) is highly repetitive in the genome and transcription occurs from several sites both within the same chromosome and within the entire genome. Treatment with the growth factor also induced transcription of the EMT-related genes *SNAI1*, *SNAI2* (Slug), and *ZEB1*, and mesenchymal markers *VIM* (vimentin) and *ACTA2* (α-smooth muscle actin), and downregulated *CDH1* gene (encoding E-cadherin) transcription. The RAS LNA-labeled oligonucleotide abolished the observed activation of *HSAT2*, *VIM*, *ACTA2*, *SNAI1*, and *SNAI2*. However, it had little influence on ZEB1 transcription (Figure 4D). The FAS LNA-labeled oligonucleotide did not influence the transcription significantly (Figure 4D).

Taken together, our data suggest that HSAT2 transcripts can facilitate the process of EMT in epithelial cancer cells.

## 3. Discussion

### 3.1. Genomic Mapping of the HSAT2 Transcript 

Tandemly repeated satellite DNA was first described more than 60 years ago as additional DNA bands in cesium density gradient ultracentrifugation experiments [2,35]. However, not only its functions but also the exact organization of its sequence remained obscure until recently. However, despite significant advances in this area, we still do not know much about the structure and functions of these amazing sequences. One of the main obstacles to the study of the functions of tandemly repeated satellite DNA was the lack of a complete map of the chromosome pericentromeric regions [20]. Until 2022, HSAT1–3 arrays in the human genome were not mapped precisely with their approximate locations in the genome assembly marked as enormous gaps filled with placeholder “N” characters [7]. The situation changed drastically when a new gapless genome assembly, CHM13-T2T, with fully assembled pericentromeres and centromeres, was published [21,24]. Satellite DNAs have been annotated in the assembly. The new assembly gives new possibilities to all those who work with centromeric and pericentromeric sequences.

In the first step of our study, we mapped the satellite DNA transcript previously described in our laboratory onto the human genome using the CHM13-T2T assembly. The transcript was originally described as an HSAT3 transcript originating from chromosome 1 [13] and later as an HSAT3 transcript from the human preovulatory oocyte transcriptome [17]. However, according to new satellite annotations in the CHM13-T2T assembly, we can undoubtedly define it as an HSAT2 sequence (Figure 1C and Figure 2). The sequences of HSAT2 and HSAT3 are closely related and originated from the same pentameric repeat, though HSAT2 is more diverged and based on 23 or 26 units [4]. Before the publication of the CHM13-T2T assembly, both satellites were sometimes referred to as HS2/3 or HSII/HSIII [36,37] due to the lack of a proper bioinformatic/sequencing approach and their short irregular nature. The gapless assembly where satellites are annotated using the K-mer approach gives a possibility for precise classification of a sequence. All the hits for the cloned sequence were mapped onto HSAT2 arrays that were absent in the previous assembly. The sequence was the most abundant on chromosomes 1, 10, and 16 (Table 1). Arrays on chromosomes 1 and 10 share a high degree of similarity [7]; therefore, this result can be expected at least for chromosomes 1 and 10.

According to our data, the sequence of the cloned transcript was tandemly repeated but the arrays were often oriented in opposite directions, being positioned on the ‘plus’ or ‘minus’ strand in the CHM13-T2T assembly (Table 1, Figure 2A,B). Inversions of centromeric and pericentromeric satellite arrays including HSAT2 were demonstrated by Karen Miga’s team [24]. Taking their study into account, mapping the transcript onto different DNA strands is expectable. However, the strand-specificity of satellite DNA transcription was demonstrated by several laboratories including our own [10,13,16,17], and is also shown in our study where only the RAS oligonucleotide (complementary to G-strand) abolished endogenous transcription of HSAT2 (Figure 4D). The HSAT strands were defined as C-strand (built on ATTCC monomer and its variations) and G-strand (GGAAT and variations) [6,38,39]. At that time, inversions of satellites were not demonstrated. Now, when inversions have been demonstrated, we would suggest speaking about G- or C-transcripts instead of strand specificity because the same transcripts can originate from different strands.

In our study (Figure 4), we observe strand specificity after the overexpression and induction of endogenous HSAT2 transcription by TGFβ1. However, the effects of transcription were blocked by the opposite oligonucleotide. Overexpression effects were decreased when the FAS (complementary to ATTCC transcripts) oligonucleotide was co-transfected, while TGFβ1-induced transcription was downregulated in the presence of the RAS (complementary to GGAAT) oligonucleotide. Our results suggest that endogenous HSAT transcription after TGFβ1 treatment occurs mostly from G-rich sequences in the genome. Similar results on the predominance of the G-rich genomic sequences were previously reported for the heat-shocked cancer cell line HeLa, non-stressed A431 cancer cell line, senescent fibroblasts, and oocytes [13,17,39]. However, transcripts originating from the C-rich strand have a similar effect according to our data—when a C-strand sequence is cloned under a potent promotor and is thus expressed, EMT transition is also induced. Therefore, we hypothesize that it is not sequence specificity that is important but, most likely, endogenous transcription initiation sites provide predominant transcription of the G-strand. Both variants of pentameric transcripts would have a physiological effect if both were available. However, in the nucleus, predominantly G-sequences are available for transcription probably due to promoting site localization. However, the mechanisms of HSAT transcription initiation and regulation are still poorly understood, though some regulatory sequences have been identified [40]. The analysis of HSAT and HSAT-adjacent regions is facilitated by the new human genome assembly, and thus new data on regulatory sequences within HSAT or their neighborhood might be expected.

### 3.2. The Influence of HSAT2 Transcription on EMT

Despite the initial ideas about transcriptionally silent pericentromeres, we know now that the DNA of all HSAT families (HSAT1-3) can be transcribed in a cell under certain circumstances (stress, malignization, cancer-associated polarization, differentiation, senescence, oogenesis, and embryogenesis) [19,25,41,42,43]. In cancer cells, HSAT DNA and RNA contribute to genetic instability [20,44,45,46,47]. In tumors as a whole, pericentromeric transcripts transcribed in microenvironment cells (e.g., fibroblasts) can increase drug resistance, provoke inflammation, activate the innate immune system, etc. [19,26,27,41]. HSAT transcription is correlated with poor prognosis and lower survival [26,48,49] and is more prominent in advanced tumors [19]. However, the mechanisms underlying the correlation are not clear yet. HSAT 2 and 3 transcripts influence the tumorigenic process in multiple ways, by modifying cancer cells and their microenvironment. In the present study, we report another possible aspect of HSAT2 transcription that might be important for patient survival. According to our data, HSAT2 transcripts might be involved in EMT increasing SNAI1/SNAI2 transcription (Figure 4A–D). EMT is a dynamic and reversible process through which epithelial cells present a mesenchymal-like phenotype, which is defined by changes in cell markers, morphology, and migration functions. It promotes cancer cell invasion and migration [50,51]. A high correlation between invasion and metastasis and the loss of E-cadherin has been demonstrated [52]. SNAI1 protein induces a complete EMT, while ZEB1 is an EMT stimulator whose gene activity is induced by SNAI1 [50,53]. It is suggested that ZEB1 RNA is more stable than SNAI1 RNA, and thus ZEB1 could possibly prolong the repression of epithelial genes initiated by Snail [53]. The stability of *ZEB1* RNA might explain the absence of *ZEB1* downregulation after the inactivation of HSAT2 (Figure 4D). EMT can be induced by different factors (TGFβ1, TNFα, etc.) secreted by different cells in a tumor. However, it is clear now that lncRNAs play important roles in the EMT progression at least in pancreatic ductal adenocarcinoma (summarized in [50]). Two recent studies performed by Dr. D. Ting’s team have reported HSAT2 lncRNA’s possible role in EMT in pancreatic ductal adenocarcinoma and ovarian cancer [49,54]. Our data give evidence of the involvement of HSAT2 transcription in EMT in lung cancer cells. The effect was less prominent in cervical cancer HeLa cells—we observed SNAI1/SNAI2 upregulation (though not so prominent as in A549 cells) in HeLa cells overexpressing HSAT2 without significant changes in cell morphology (Figure 4A,B). It was assumed earlier that EMT is an ‘all-or-nothing’ process. However, it is proven now that EMT does not necessarily present as a binary switch between two extremes yet can render cells a partial or hybrid phenotype that simultaneously holds epithelial and mesenchymal characteristics. This new concept, recently defined as epithelial–mesenchymal plasticity (EMP), grants cancer cells a higher adaptive potential depending on microenvironmental cues [55].

## 4. Materials and Methods

### 4.1. Cell Cultures 

A549 (RRID:CVCL_0023 at https://www.cellosaurus.org, accessed on 28 February 2023) and HeLa (M strain, HeLa M (RRID:CVCL_R965) at https://www.cellosaurus.org, accessed on 28 February 2023) cells were obtained from the shared research facility “Vertebrate cell culture collection” supported by the Ministry of Science and Higher Education of the Russian Federation (Agreement No. 075-15-2021-683). Cells were grown in a Dulbecco’s modified Eagle’s low glucose medium (DMEM LG GlutaMAX, ThermoFisher Scientific, Waltham, MA, USA) supplemented with 10% FBS (fetal bovine serum; HyClone, Logan, UT, USA), 100 U/mL penicillin, and 100 μg/mL streptomycin (Life Technologies, Carlsbad, CA, USA). The cells were seeded into a flask (TPP, Trasadingen, Switzerland) and were further grown at 37 °C in a humidified 5% CO_2_ atmosphere. The medium was changed every 3 days. Cells that reached 70–80% confluence were subcultured using standard methods.

EMT was induced by TGFβ1. The growth factor TGFβ1 (5 ng/mkL, Himedia, India) was added for 48 h to A549 or HeLa cells that were grown in a serum-free DMEM LG medium for 24 h before the incubation with TGFβ1. The cells were then harvested for RNA extraction followed by reverse transcription and qPCR.

### 4.2. Oligonucleotides

The primers and oligonucleotides (Synthesized by Lumiprobe, Moscow, Russia, Evrogen, Moscow, Russia, and DNK-sintez, Moscow, Russia) used in the study are given in Table 2.

### 4.3. Computational Analysis

The human T2T-CHM13v2.0 genome (https://www.ncbi.nlm.nih.gov/assembly/GCF_009914755.1, accessed on 21 February 2023) was used to identify transcript localization. A notebook for its reproduction is available in the GitHub repository (https://github.com/zilov/hsat2_transcript_analysis, accessed on 21 February 2023). Mapping of the transcript sequence on the genome was performed using the standalone version of BLAST v2.12.0+ [56] (-task blastn -perc_identity 80). The resulting mapping results were converted to BED format using blast2bed (https://github.com/nterhoeven/blast2bed, accessed on 21 February 2023). USCS Genome Browser (human genome T2T-CHM13v2.0 GCA_009914755.4) was used to visualize the localization of the transcript sequence on chromosomes [22].

### 4.4. Cloning

The HSAT2 sequence we had previously found in human transcriptomes [17] was amplified from human genomic DNA. The following primers were used in PCR (Table 2): AgeI-2F (5′—GCT ACC GGT CGA TTC TGT TCG GTG ATT CC—3′) and SalI-1R (5′—CGT CGA CTG CTG AAA TCC AAT ATG ATC ATC ATC GAA—3′). The forward primer, AgeI-HS2F, contained a restriction site for AgeI restrictase; the reverse primer, SalI-HS2R, contained a restriction site for SalI restrictase. The PCR product was cloned into the cloning vector pAL2-T (Evrogene, Russia; plasmid # TA002) using T4DNA ligase (Evrogene, Moscow, Russia). The cloned fragment sequence verification was performed using M13 sequencing primers (M13F 5′ GTA AAA CGA CGG CCA GTG 3′ and M13R 5′-GGA AAC AGC TAT GAC CAT G-3′) on an ABI Prism 3100 Genetic Analyzer (Applied Biosystems). The *E. coli* strain DH5α was transformed with the obtained HSAT2-pAL2 plasmid, and the plasmid was purified. Mammalian expression vector pTurboGFP-c (Evrogene, plasmid # FP511) was digested with AgeI and SalI restriction endonuclease to remove the TurboGFP gene. The cloned HSAT2 sequence was excised from the HSAT2-pAL2- plasmid using restriction endonuclease AgeI and SalI. The sequence of interest (HSAT2) was separated from the vector in a 1% agarose gel, excised from the gel, and ligated with a pTurboGFP-c expression vector that was treated as described above. Several clones were obtained, and their plasmid inserts were sequenced (see the Results Section 2). The obtained HSAT2-pTurbo-C plasmid (plasmid p4.1 containing the insert with Acc No OP912406.2) was cloned in *E. coli* DH5a strain. The plasmid was purified from the bacteria using a Plasmid Midiprep 2.0 kit (Evrogen, Moscow, Russia) and used for transfection.

### 4.5. Transfection

Cells (A549 and HeLa) were grown in 6-well plates until the cells were 70–75% confluent. The growing medium was then changed to Opti-MEM™ medium for 1 h. HSAT2-pTurbo-C plasmid (2 mkg/well) was used for transfection with Lipofectamine™ 3000 (Thermofisher, Waltham, MA, USA). Cells were incubated with the plasmid and Lipofectamine™ 3000 for 14 h, then the medium was changed to low glucose medium (DMEM LG GlutaMAX, ThermoFisher Scientific, Waltham, MA, USA) supplemented with 10% FBS. After 48 h, cells were harvested and used either for RNA extraction or for RNA-FISH.

In the overexpression knockdown experiments, cells were co-transfected with HSAT2-pTurbo-C plasmid (2 mkg/well) and the FAS or RAS oligonucleotide (at a final concentration of 100 nM) (Table 2). To access the influence of sense and antisense oligonucleotides on endogenous HSAT2 transcription induced by TGFβ1, cells were transfected with the FAS or RAS oligonucleotide (100 nM) 24 h prior to adding TGFb (5 ng/mL). The oligonucleotides were modified (Table 2): four nucleotides at the 3′ and 5′ ends were 2-o-methylated while the remaining core nucleotides were locked nucleic acid (LNA)-nucleotides. Modification of 2′-O-methyl increases the resistance of RNA to the effects of nucleases, significantly increases its affinity to the target RNA (melting point, compared with the DNA–RNA duplex is higher), and ensures binding of RNase H to the sites of duplexes [57,58]. We compared the HSAT2 knockdown efficacy of LNA and 2-o-Me+LNA oligonucleotides (Supplementary Figure S3). According to the qPCR data, 2-o-Me+LNA oligonucleotides were more effective in HSAT2 knockdown experiments. Therefore, the oligonucleotides end-labeled with 2-o-Me and simultaneously core modified with LNA were chosen for the knockdown experiments.

### 4.6. PCR and qPCR

Cells were harvested and used for RNA extraction with an RNA-solo kit (Evrogen, Russia). cDNA was reverted using Mmulv revertase (Evrogen, Russia), and either oligo-dT, M13-oligo-dT (CAggAAACAgCTATgACTTTTTTTTTTTTN), or HSAT2 + or − strand-specific oligonucleotides (specified in the figure legends). Quantitative PCR reactions were performed in a Bio-Rad CFX96 real-time system (Hercules, California, United States) using PowerTrack ™ SYBR Green Master Mix (Thermofisher, Waltham, MA, USA) with specific primers listed in Table 2. The thermocycling conditions were as follows: 95 °C for 5 min, followed by 40 cycles at 95 °C for 15 s, and 60 °C for 30 s. A final heating step of 65 °C to 95 °C was performed to obtain melting curves of the final PCR products. mRNA expression levels were calculated by the 2^−ΔΔCt^ method [59] with the levels of gene transcription normalized to the housekeeping genes *GAPDH* encoding glyceraldehyde 3-phosphate dehydrogenase (GAPDH). Data are shown as the mean ± SD.

### 4.7. Fluorescent In Situ Hybridization (FISH)

For DNA–DNA FISH (i.e., a DNA probe hybridized to target DNA), fixed preparations of metaphase chromosomes were denatured in a hybridization mixture (70% formamide in 2 × SSC) for 2 min at 74 °C and were then dehydrated in three changes of ethanol (70%, 80%, and 96%) and air-dried. A probe was Cy3-labelled by PCR with 2F/1R primers set. The probe (1 µg/mL) was denatured in the hybridization mix Hybrisol VII (MP Biomedicals, Santa Ana, CA, USA) and applied on slides. Hybridization was carried out overnight in a humidified chamber at 37 °C. After hybridization, the slides were washed in 50% formamide in 2 × SSC for 5 min at 41 °C, then at room temperature, in 1 × SSC and 0.5 × SSC for 5 min. Then, the slides were rinsed in H_2_O mounted in a medium, containing DAPI and antifade reagent (Invitrogen, Thermofisher, Waltham, MA, USA).

The sites of HSAT2 transcript localization were detected with a Cy3-labelled oligonucleotide DNA probe Tr-3′ (Table 2) in DNA–RNA FISH (i.e., a DNA probe hybridized to target RNA). Cells grown on coverslips were fixed with 2% paraformaldehyde in PBS and permeabilized with 0.1% Triton X_100. Then (omitting denaturation), hybridization mix Hybrisol VII (MP Biomedicals) containing Cy3-Tr-3′ (1 µg/mL) was applied to cells. The hybridization and washing steps were carried out as described above for FISH with a 2F/1R probe.

### 4.8. Confocal Microscopy 

The images were acquired with an Olympus FV3000 confocal microscope (Olympus, Tokyo, Japan). To detect DAPI and Cy3, the 405 and 561 nm diode lasers were used for excitation, respectively.

### 4.9. Statistics

The data are representative of three or more independent experiments. qPCR was performed in three technical and three biological replicates. GraphPad Prism version 8.0.0 for Windows (GraphPad Software, San Diego, CA, USA) was used. Results are reported as the mean and SD. ANOVA followed by Sidak’s multiple comparisons test was used in qPCR studies. Significant difference was assessed with a *p*-value < 0.05.

## 5. Conclusions

Our results suggest that the HSAT2 transcripts sequence can be mapped in both orientations onto chromosomes 1, 2, 7, 9, 10, 16, and 17, being the most abundant on chromosomes 1, 10, and 16. Transcription of the HSAT2 sequence can induce EMT in some cancers.

## Figures and Tables

**Table 1:** Mapping of the cloned transcript onto T2T-CHM13 assembly.

Chromosome	Total Number of Hits	CEN Satellite DNA Annotation *	Number of Hits	Strand	Chromosome Band
chr1	7876	hsat2_1_1(B)	3	−	1q12
		hsat2_1_3(A1,B)	411	+	1q12
		hsat2_1_4(B,A1,A2)	23	+	1q12
		hsat2_1_5(B)	2	+	1q12
		hsat2_1_6(A2,A1,B)	7437	−	1q12
chr2	118	hsat2_2_2(B)	9	+/−	2p11.2
		hsat2_2_3(B)	5	+/−	2p11.2
		hsat2_2_4(B)	10	+/−	2p11.2
		hsat2_2_5(B)	76	+/−	2p11.2
		hsat2_2_6(B)	8	+	2p11.2
		hsat2_2_7(B)	10	−	2q11.2
chr7	83	hsat2_7_5(B)	10	+/−	7p11.2
		hsat2_7_6(B)	6	+	7p11.2
		hsat2_7_7(B)	6	−	7p11.2
		hsat2_7_10(B)	7	+	7p11.2
		hsat2_7_12(B)	2	−	7p11.2
		hsat2_7_13(B)	14	+	7p11.2
		hsat2_7_15(B)	3	−	7p11.2
		hsat2_7_16(B)	6	−	7p11.2
		hsat2_7_17(B)	18	+	7p11.2
		hsat2_7_18(B)	10	−	7p11.2
		hsat2_7_19(B)	1	+	7q11.21
chr9	10	hsat2_9_1(B)	10	+	9p11.2
chr10	273	hsat2_10_1(B)	6	−	10p11.21
		hsat2_10_2(A1,A2)	9	+	10q11.21
		hsat2_10_3(A1,A2)	247	−	10q11.21
		hsat2_10_4(B)	1	−	10q11.21
chr16	3833	hsat2_16_3(B)	1	+/−	16p11.2
		hsat2_16_4(B)	4	+	16p11.2
		hsat2_16_5(B)	10	−	16q11.2
		hsat2_16_6(B)	4	−	16q11.2
		hsat2_16_7(B)	9	+/−	16q11.2
		hsat2_16_8(B)	2	−	16q11.2
		hsat2_16_9(B)	12	−	16q11.2
		hsat2_16_10(B)	1	−	16q11.2
		hsat2_16_11(B)	11	−	16q11.2
		hsat2_16_12(B)	12	−	16q11.2
		hsat2_16_13(B)	2	−	16q11.2
		hsat2_16_15(B)	3765	+/−	16q11.2
chr17	31	hsat2_17_1(B)	2	+	17p11.2
		hsat2_17_2(B)	29	+	17p11.2
chr22	48	hsat2_22_2(B)	4	+	22q11.21
		hsat2_22_3(B)	17	+	22q11.21
		hsat2_22_4(B)	18	+	22q11.21
		hsat2_22_5(B)	8	−	22q11.21
chrY	1	ct_Y_48	1	+	q11.21
Total			12,262		

*—the names of the HSAT subfamilies (HSAT family_Chromosome No_HSAT subfamily) in CEN satellite DNA column annotation are given according to the CEN satellite DNA annotation track in the T2T-CHM13 assembly. The pipeline for the HSAT definition is given in [24,29]. The sequence orientation is shown in the strand column. ‘+’—all the hits were mapped onto a C-rich strand, ‘−’—all the hits were positioned in a G-rich strand, ‘+/−’—the hits within the subfamily were mapped onto the chromosomes bidirectionally.

**Table 2:** Oligonucleotides used in the study.

Oligonucleotide	Sequence	Target	Reference	Application
2F	CGATTCTGTTCGGTGATTCC	BK063198 OP912407.2 OP912406.2 OP912405.2 OP912404.2 OP912403.2	Own design	qPCR
1R	TGAAATCCAATATGATCATCATCGAA			qPCR
2R	AATGGATGGACTCATCATCG	With 2F—5′ end of OP912406.2	Own design	qPCR
1F	CGTTTCCTTTCGATGGCGTT	With 1R—3′ end of OP912406.2	Own design	qPCR
S1	AGTCCATTCAATGATTCCATTCCAGT	HSAT2 transcript *** X60726.1	[13]	qPCR
AS1	AATCATCATCCAACGGAAGCTAATG			qPCR
SnailF	CTCTTTCCTCGTCAGGAAGC	*SNAI1* NM_005985.4	[30]	qPCR
SnailR	GGCTGCTGGAAGGTAAACTC			qPCR
SlugF	ATGAGGAATCTGGCTGCTGT	*SNAI2* NM_003068.5	[30]	qPCR
SlugR	CAGGAGAAAATGCCTTTGGA			qPCR
Zeb1F	GTCAGCCCTGCAGTCCAAGAACCAC	*ZEB1* NM_001128128.3	[19]	qPCR
Zeb1R	CCGCATTTTCTTTTTGGGCGGTGTA			qPCR
VimF	GGCTCAGATTCAGGAACAGC	*VIM* NM_003380.5	[31]	qPCR
VimR	AGCCTCAGAGAGGTCAGCAA			qPCR
Col11A1F	GACTATCCCCTCTTCAGAACTGTTAAC	*COL11A1* NM_001854.4	[19]	qPCR
Col11A1R	CTTCTATCAAGTGGTTTCGTGGTTT			qPCR
Col1A1F	TCAGCAAGAACCCCAAGGACAAGAGG	*COL1A1* NM_000088.4		qPCR
Col1A1R	AGGAAGGTCAGCTGGATGGCCACAT			qPCR
eCadF	GCTGGACCGAGAGAGTTTCC	*CDH1* NM_004360.3	Own design	qPCR
eCadR	CGACGTTAGCCTCGTTCTCA			qPCR
AgeI-2F *	*GCTACCGGT*CGATTCTGTTCGGTGATTCC	OP912406.2		Cloning into an expression vector
SalI-1R *	*CGTCGACTGC*TGAAATCCAATATGATCATCATCGAA			
GAPDHF	AGGTCGGAGTCAACGGATTT	NM_002046.7	Own design	Reference gene for qPCR
GAPDHR	TTCCCGTTCTCAGCCTTGAC			
FAS **	meAmeAmeUmeGlnGlnAlnAlnUlnClnGlnAlnAlnUlnGlnGmeAmeAmeUmeC	OP912407.2 OP912406.2 OP912405.2 OP912404.2 OP912403.2 X60726.1	Own design	HSAT2 2F/1R cloned transcript knockdown
RAS **	meGmeAmeUmeUlnClnClnAlnUlnUlnClnGlnAlnUlnUlnCmeCmeAmeUmeU		Own design	HSAT2 2F/1R cloned transcript’s reverse strand knockdown
Scr	meAmeAmeUmeUmeCmeUmeCmeCmeGmeAmeAmeCmeGmeUmeGmeUmeCmeAmeCmeGmeU	Scramble sequence for knockdown	[32]	A control sequence in knockdown experiments
Tr-3′	Cy3—GATGGCGTTTCCATTCGATTCCATTCGATGTTGATTCCATTTGTTTCCATTGGATGATGATTCCGTTCGTGTCCATTCGA	BK063198, OP912407.2 OP912406.2 OP912405.2 OP912404.2 OP912403.2	Own design	RNA-FISH

*—an adaptor sequence containing correspondent restriction site is shown in italics. **—me-2-o-Me modification, ln—LNA modification. ***—originally described as HS3. In the T2T-CHM13 assembly, the sequence is annotated as HSAT2.

## Data Availability

The cloned sequences reported here have been deposited in GenBank (GenBank accession numbers: OP912407.2, OP912406.2, OP912405.2, OP912404.2, and OP912403.2). The sequence we annotated in the human genome [17] as HSAT2 transcript is available in the Third Party Annotation Section of the DDBJ/ENA/GenBank databases under the accession number TPA: BK063198. The results of the bioinformatic analysis and a notebook for its reproduction are available in the GitHub repository https://github.com/zilov/hsat2_transcript_analysis, accessed on 21 February 2023.

## Supplementary Materials

The following supporting information can be downloaded at: https://www.mdpi.com/article/10.3390/ijms24086918/s1.
