# Supplementary material for: Overexpression of Pericentromeric HSAT2 DNA Increases Expression of EMT Markers in Human Epithelial Cancer Cell Lines
Source: Int J Mol Sci. 2023 Apr 7;24(8):6918. doi: 10.3390/ijms24086918 (PMC10138405; doi:10.3390/ijms24086918)
Supplement: Supplementary file 1 [file ijms-24-06918-s001.zip › Supplementary Figures.pdf]

Overexpression of pericentromeric HSAT2 DNA increases expression of EMT markers in human epithelial cancer cell lines

Nikita Ponomartsev[1*#], Danil Zilov [1,2#], Ekaterina Gushcha [1], Alexandra Travina[1], Alexander Sergeev[1], Natella Enukashvily [1*].

[1]Institute of Cytology, Russian Academy of Sciences, St. Petersburg, 194064, Russia;

[2]Applied Genomics Laboratory, SCAMT Institute, ITMO University, Saint Petersburg 197101, Russia;

# - Two authors have contributed equally

*Correspondence: n.enukashvily@incras.ru (N.E.), ponomartsev@yandex.ru (N.P.)

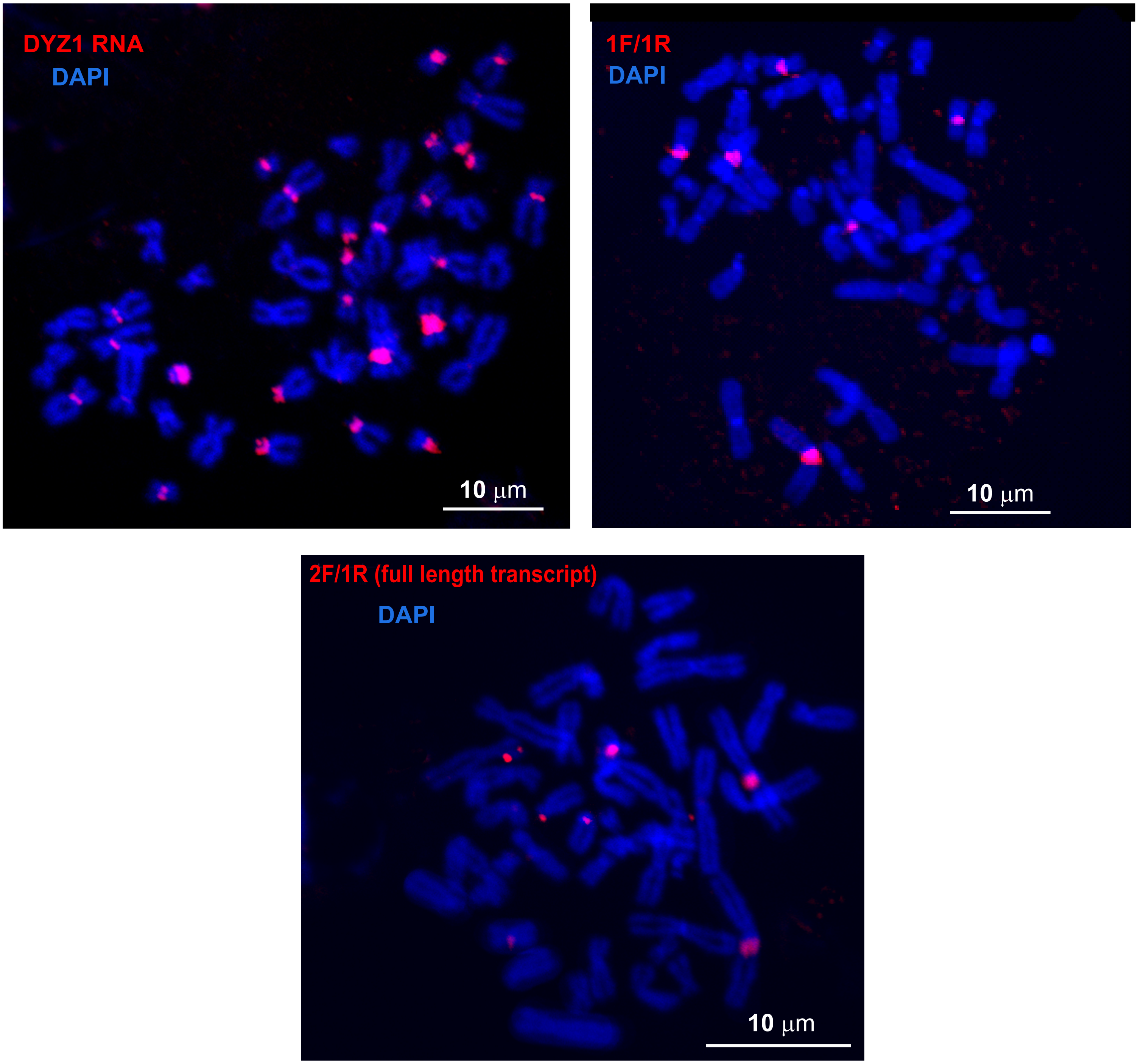

Supplementary Figure S1. Pericentromeric localization of DYZ1, 1F/1R and 2F/1R sequences probed revealed by FISH. Pericentromeric location of DYZ1 (used for fishing out 2F/1R RNA from transcriptome in our previous work [17 in the main text]), 1F/1R (cDNA 5') and 2F/1R (a full length transcript registered with TPA Acc No Bk063198) on mitotic chromosomes of human umbilical cord mesenchymal stem cell obtained as described in our previous publications (DOI: https://doi.org/10.1016/j.ejphar.2020.173182 and https://doi.org/10.17816/mechnikov201810111-19). All probes are shown in red. Chromosomes are counterstained with DAPI (blue). Scale bars are shown in the images.

Overexpression of pericentromeric HSAT2 DNA increases expression of EMT markers in human epithelial cancer cell lines

Nikita Ponomartsev[1*#], Danil Zilov [1,2#], Ekaterina Gushcha [1], Alexandra Travina[1], Alexander Sergeev[1], Natella Enukashvily [1*].

1 Institute of Cytology, Russian Academy of Sciences, St. Petersburg, 194064, Russia;

2 Applied Genomics Laboratory, SCAMT Institute, ITMO University, Saint Petersburg 197101, Russia;

# - Two authors have contributed equally

* Correspondence: n.enukashvily@incras.ru (N.E.), ponomartsev@yandex.ru (N.P.)

```
2F/1R     -------TTTTTTTTTTTTTTTTTTTTTTTTTTTTCGATGATTGTTCCATTCGATTCTG 53
HS1.1     ATATA-AGCAGAGCTGGTTTAGTGAACCGTCAGATCCGCTAGCGCTACCGGTCGATTCTG 118
HS1.2     ATATATAGCAGAGCTGGTTTAGTGAACCGTCAGATCCGCTAGCGCTACCGGTCGATTCTG 120
HS1.3     ATATAATGCAGAGCTGGTTTAGTGAACCGTCAGATCCGCTAGCGCTACCGGTCGATTCTG 120
HS4.1     ATATAATGCAGAGCTGGTTTAGTGAACCGTCAGATCCGCTAGCGCTACCGGTCGATTCTG 120
HS4.2     ATATAA-GCAGAGCTGGTTTAGTGAACCGTCAGATCCGCTAGCGCTACCGGTCGATTCTG 119
                        *  ***  *      *    * ** *     * **  *********

2F/1R     TTCGGTGATTCCATTCGATTCCATTTGATAATGATTCCGTTCGAGACCATTCGATGATTC 113
HS1.1     TTCGGTGATTCCATTCGACTTAATTTGATAATGATTCCATTCGAGACCATTCGATGATTC 178
HS1.2     TTCGGTGATTCCATTCGACTTAATTTGATAATGATTCCATTCGAGACCATTCGATGATTC 180
HS1.3     TTCGGTGATTCCATTCGACTTAATTTGATAATGATTCCATTCGAGACCATTCGATGATTC 180
HS4.1     TTCGGTGATTCCATTCGATTCCATTTGATAATGATTCCAATCGAGACCATTCGATGATTC 180
HS4.2     TTCGGTGATTCCATTCGATTCCATTTGATAATGATTCCAATCGAGACCATTCGATGATTC 179
          ****************** *  ****************  ********************

2F/1R     CATTCAATTCCATTCAATAAAGATTCCATTCGAGTCCATTCATTGATTCCCTTCAAGTCC 173
HS1.1     TATTCAATTCCATTCAATAATGATTCCATTCGAGTCCATTCAACGATTCCATTCAAGTCC 238
HS1.2     TATTCAATTCCATTCAATAATGATTCCATTCGAGTCCATTCAACGATTCCATTCAAGTCC 240
HS1.3     TATTCAATTCCATTCAATAATGATTCCATTCGAGTCCATTCAACGATTCCATTCAAGTCC 240
HS4.1     CATTCAATTCCATTCAATAATGATCCCTTTCGAGTCCATTCAATGATTCCATTCCAGTCC 240
HS4.2     CATTCAATTCCATTCAATAATGATCCCTTTCGAGTCCATTCAATGATTCCATTCCAGTCC 239
           ******************* *** ** **************  ****** *** *****

2F/1R     ATTCGATGATTCCATCAGATTCCATTCAATGAATCCATTCGATTCCATTCAGTGATGATT 233
HS1.1     ATTCGATGATTCCATCTGATTCCATTCAATGAATCCATGCGATTCCATTCTATGATGATT 298
HS1.2     ATTCGATGATTCCATCTGATTCCATTCAATGAATCCATGCGATTCCATTCTATGATGATT 300
HS1.3     ATTCGATGATTCCATCTGATTCCATTCAATGAATCCATGCGATTCCATTCTATGATGATT 300
HS4.1     ATTCGATGATTCCATCTGATTCCATTCAATGAATCCATTCGATTCCATTCTATGAGGACT 300
HS4.2     ATTCGATGATTCCATCTGATTCCATTCAATGAATCCATTCGATTCCATTCTATGAGGACT 299
          **************** ********************* ***********  *** ** *

2F/1R     CCATTCATTTCCATCTGATGATGATTCCATTCGATTCCATTCAATGATTCCATTCAATTC 293
HS1.1     CCATTCATTTCCATCTGATGATGATTCCATTCGATTCCATTCAATGATTCCTTTCGATTC 358
HS1.2     CCATTCATTTCCATCTGATGATGATTCCATTCGATTCCATTCAATGATTCCTTTCGATTC 360
HS1.3     CCATTCATTTCCATCTGATGATGATTCCATTCGATTCCATTCAATGATTCCTTTCGATTC 360
HS4.1     CCATTCATTTCCATCTGATGATGATTCCATTCGATTCCATTCAATGATACCATTCGATTC 360
HS4.2     CCATTCATTTCCATCTGATGATGATTCCATTCGATTCCATTCAATGATACCATTCGATTC 359
          ************************************************ ** *** ****

2F/1R     CATTTGATGATGATTTCAATCAATTTCATTCGGTGATTCCATTCGAATCCACTCGATGAT 353
HS1.1     CATTTGATGATGATTTCAATCAATTTCATTCGGTGATTCCACTCGAATCCACTCGATGAT 418
HS1.2     CATTTGATGATGATTTCAATCAATTTCATTCGGTGATTCCACTCGAATCCACTCGATGAT 420
HS1.3     CATTTGATGATGATTTCAATCAATTTCATTCGGTGATTCCACTCGAATCCACTCGATGAT 420
HS4.1     CATTTGATGATGATTTCAATCAATTTTATTCGATGATTCCATTCGAATCCATTCGATGAT 420
HS4.2     CATTTGATGATGATTTCAATCAATTTTATTCGATGATTCCATTCGAATCCATTCGATGAT 419
          ************************** ***** ******** ********* ********

2F/1R     GAGTCCATCCATTTCAATTTCATGATAATTCCATTCGTTTCCTTTCGATGGCGTTTCCAT 413
HS1.1     GAGTCCATCCATTTCAATTTCATGATAATTCCATTCGTTTCCTTTCGATGGCGTTTCCAT 478
HS1.2     GAGTCCATCCATTTCAATTTCATGATAATTCCATTCGTTTCCTTTCGATGGCGTTTCCAT 480
HS1.3     GAGTCCATCCATTTCAATTTCATGATAATTCCATTCGTTTCCTTTCGATGGCGTTTCCAT 480
HS4.1     GAGTCCATCCATTTCAATTTCATGATAATTCCATTCGTTTCCTTTCGATGGCGTTTCCAT 480
HS4.2     GAGTCCATCCATTTCAATTTCATGATAATTCCATTCGTTTCCTTTCGATGGCGTTTCCAT 479
          ************************************************************

2F/1R     TCGATTCCATTCGATGTTGATTCCATTTGTTTCCATTGGATGATGATTCCGTTCGTGTCC 473
HS1.1     TCGATTCCATTCGATGTTGATTCCATTTGTTTCCATTGGATGATGATTCCGTTCGTGTCC 538
HS1.2     TCGATTCCATTCGATGTTGATTCCATTTGTTTCCATTGGATGATGATTCCGTTCGTGTCC 540
HS1.3     TCGATTCCATTCGATGTTGATTCCATTTGTTTCCATTGGATGATGATTCCGTTCGTGTCC 540
HS4.1     TCGATTCCATTCGATGTTGATTCCATTTGTTTCCATTGGATGATGATTCCGTTCGTGTCC 540
HS4.2     TCGATTCCATTCGATGTTGATTCCATTTGTTTCCATTGGATGATGATTCCGTTCGTGTCC 539
          ************************************************************

2F/1R     ATTCGATGATGATCATATTGGATTTCATTCCATAATTCTATTCGAATCCATTTGATGAT- 532
HS1.1     ATTCGATGATGATCATATTGGATTTCAGCAGTCGACGGTACCGCGGGCCCGGGATCCACC 598
HS1.2     ATTCGATGATGATCATATTGGATTTCAGCAGTCGACGGTACCGCGGGCCCGGGATCCACC 600
HS1.3     ATTCGATGATGATCATATTGGATTTCAGCAGTCGACGGTACCGCGGGCCCGGGATCCACC 600
HS4.1     ATTCGATGATGATCATATTGGATTTCAGCAGTCGACGGTACCGCGGGCCCGGGATCCACC 600
HS4.2     ATTCGATGATGATCATATTGGATTTCAGCAGTCGACGGTACCGCGGGCCCGGGATCCTCC 599
          ***************************       *   **       **
```

GenBank Acc No for the annotated sequence:
2F/1R - BankIt2670656 HSAT2 Bk063198
GenBank Acc No for cloned sequences:
HS1.1 - OP912403.2 ,
HS1.2 - OP912404.2,
HS1.3 - OP912405.2,
HS4.1 - OP912406.2,
HS4.2 - OP912407.2

Supplementary Figure S2. Alignment of 5 cloned sequences and the HSAT2 transcript's sequence (2F/1R) from assembled transcriptome. The sequence annotated in our previous work (registered with Acc No BankIt2670656 HSAT2 Bk063198) was used for 2F/1R primer set design. Genomic DNA was amplified using 2F/1R primers set (see Table 2 of the main text). The amplified fragments (HS1.1, HS1.2, HS1.3, HS4.1, HS4.2) were cloned and sequenced. All the obtained clones were aligned to original annotated sequence to confirm the results of cloning. Accession numbers for all the sequences are given in the purple frame in the image. BLAST tool was used for the alignment [57 of the main text]

Overexpression of pericentromeric HSAT2 DNA increases expression of EMT markers in human epithelial cancer cell lines

Nikita Ponomartsev[1*#], Danil Zilov [1,2#], Ekaterina Gushcha [1], Alexandra Travina[1], Alexander Sergeev[1], Natella Enukashvily [1*].

1 Institute of Cytology, Russian Academy of Sciences, St. Petersburg, 194064, Russia;

2 Applied Genomics Laboratory, SCAMT Institute, ITMO University, Saint Petersburg 197101, Russia;

# - Two authors have contributed equally

* Correspondence: n.enukashvily@incras.ru (N.E.), ponomartsev@yandex.ru (N.P.)

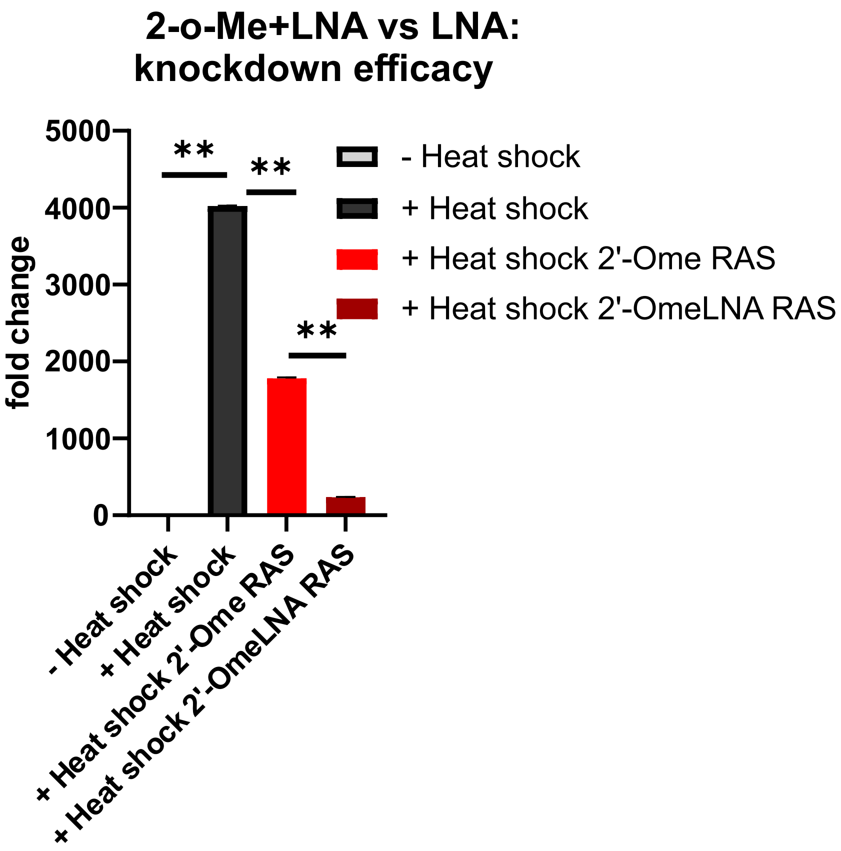

Supplementary Figure S3. Efficacy of 2-o-Me+LNA oligonucleotides and LNA oligonucleotides in knockdown experiments on heat-shocked A549 cells

Heat shock is known as the most potent stimulator of HSAT2, HSAT3 transcription. Therefore, we used the model to choose a better modification pattern of antisense oligonucleotides. A549 were subjected to heat shock (60 min, 43C). In an hour of recovery period, cells were collected and used for RNA purification, polydT cDNA synthesis and qPCR with 2F/1R primer set (see Table 2 of the main text) to quantify HSAT2 transcription in cells with (+Heat shock) and without (-Heat shock) heat shock treatment. RAS oligonucleotide (Table 2 of the main text) was modified : in 2'-OmeLNA RAS, four nucleotides at 3' and 5' ends were 2-o-methylated while the remaining core nucleotides were locked nucleic acid-(LNA)-nucleotides; in 2'-Ome RAS, all nucleotides were 2'-o-methylated. HSAT2 transcription level in cells of '-Heat shock' group (not stressed, not transfected with modified RAS) was set to 1. Transcription in other groups was quantified and measured as a fold change ratio to -Heat shock cells. ** - p<0.01.
